# A Novel HRD Signature Is Predictive of FOLFIRINOX Benefit in Metastatic Pancreatic Cancer

**DOI:** 10.1093/oncolo/oyad178

**Published:** 2023-06-24

**Authors:** Kuei-Ting Chen, Russell Madison, Jay Moore, Dexter Jin, Zoe Fleischmann, Justin Newberg, Alexa Schrock, Neeru Bhardwaj, Katherine T Lofgren, Jie He, Garrett Frampton, Priti Hegde, David Fabrizio, Michael J Pishvaian, Ericka Ebot, Aatur Singhi, Ethan Sokol

**Affiliations:** Foundation Medicine, Cambridge, MA, USA; Foundation Medicine, Cambridge, MA, USA; Foundation Medicine, Cambridge, MA, USA; Foundation Medicine, Cambridge, MA, USA; Foundation Medicine, Cambridge, MA, USA; Foundation Medicine, Cambridge, MA, USA; Foundation Medicine, Cambridge, MA, USA; Foundation Medicine, Cambridge, MA, USA; Foundation Medicine, Cambridge, MA, USA; Foundation Medicine, Cambridge, MA, USA; Foundation Medicine, Cambridge, MA, USA; Foundation Medicine, Cambridge, MA, USA; Foundation Medicine, Cambridge, MA, USA; Department of Oncology, Johns Hopkins University School of Medicine, SKCC, Washington, DC, USA; Foundation Medicine, Cambridge, MA, USA; Department of Pathology, University of Pittsburgh Medical Center, Pittsburgh, PA, USA; Foundation Medicine, Cambridge, MA, USA

**Keywords:** homologous recombination repair, pancreatic neoplasms, genomics, platinum, biomarker

## Abstract

**Background:**

Pancreatic cancer (PC) represents an aggressive disease with median overall survival (OS) of less than 1 year in the front-line setting. FOLFIRINOX and gemcitabine and paclitaxel (GP) are standard of care options for these patients; however, optimal selection of therapy is challenging.

**Methods:**

Comprehensive genomic profiling was performed on 8358 PC patients. Outcomes were available for 1149 metastatic PC patients treated with 1L FOLFIRINOX or GP. A scar-based measure of HRD was called using a machine learning-based algorithm incorporating copy number and indel features.

**Results:**

A scar-based HRD signature (HRDsig) was identified in 9% of patients. HRDsig significantly co-occurred with biallelic alterations in *BRCA1/2, PALB2, BARD1,* and *RAD51C/D*, but encompassed a larger population than that defined by *BRCA1/BRCA2/PALB2* (9% vs. 6%). HRDsig was predictive of 1L FOLFIRNOX chemotherapy benefit with doubled OS relative to gemcitabine and paclitaxel (GP) (rwOS aHR 0.37 [0.22-0.62]), including 25% of the population with long-term (2 year+) survival in a real-world cohort of patients. Less benefit from FOLFIRINOX was observed in the HRDsig(−) population. Predictive value was seen in both the *BRCA1/2/PALB2* mutant and wildtype populations, suggesting additional value to mutational profiling.

**Conclusion:**

A scar-based HRD biomarker was identified in a significant fraction of PC patients and is predictive of FOLFIRINOX benefit. Incorporating a biomarker like HRDsig could identify the right patients for platinum chemotherapy and potentially reduce FOLFIRINOX use by over 40%, minimizing toxicities with similar survival outcomes. Confirmatory studies should be performed.

## Teaser

Selection of first-line therapy for patients with pancreatic cancer is challenging. This article reports a scar-based biomarker of homologous recombination deficiency that was predictive of benefit from first-line FOLFIRINOX with a doubled median overall survival relative to gemcitabine and paclitaxel.

**Implications for Practice**

Selection of first-line therapy for pancreatic cancer patients is challenging, with the goal of maximizing survival and minimizing toxicity. We identified a scar-based biomarker of HRD (HRDsig) that is present in 9% of patients, at a greater frequency than *BRCA1/2/PALB2*. In a non-randomized retrospective analysis, HRDsig was predictive of benefit from 1L FOLFIRINOX with a doubled median overall survival relative to gemcitabine and paclitaxel (GP). Patients negative for HRDsig had less benefit on FOLFIRINOX relative to GP (OS 6.3 vs. 5.1 months). Prospective trials should examine whether a scar-based biomarker of HRD may improve optimal therapy selection.

## Introduction

Pancreatic cancer (PC) is the 4th leading cause of cancer-related death with a 5-year survival of only 11%.^1^ For patients with locally advanced or metastatic cancer, systemic chemotherapy is standard of care with FOLFIRINOX (leucovorin and fluorouracil plus irinotecan and oxaliplatin) and GP (gemcitabine and paclitaxel) both listed as preferred regimens in the NCCN guidelines for pancreatic adenocarcinoma.^2^ Clinical data suggests that patients treated with FOLFIRINOX may have modestly better outcomes than GP, but higher rates of adverse events on FOLFIRINOX can make this therapy less desirable. Biomarkers for FOLFIRINOX selection are still being explored and currently, the NCCN guidelines list *BRCA1/2* and *PALB2* alterations as a rationale for selecting a platinum-based regimen of FOLFIRINOX or gemcitabine + cisplatin rather than GP. It is not known if genes beyond *BRCA1/2* and *PALB2* may contribute to FOLFIRINOX sensitivity.

In ovarian and breast cancer, homologous recombination deficiency (HRD) is associated with response to platinum-based chemotherapies. Since FOLFIRINOX includes the platinum-containing agent oxaliplatin,^3^ we hypothesized that PC patients with tumors positive for an HRD signature might derive greater benefit from FOLFIRINOX relative to GP. We recently described the development of a scar-based measure of HRD (HRDsig)^4^ and sought to understand its predictive value in PC.

## Methods

### Comprehensive Genomic Profiling

Comprehensive genomic profiling (CGP) using a hybrid-capture approach targeting at least 324 genes was performed in a Clinical Laboratory Improvement Amendments (CLIA)-certified, CAP (College of American Pathologists)-accredited laboratory (Foundation Medicine Inc.) on 8358 PC patients assessable for HRD biomarkers (genomic alterations, gLOH, HRDsig).^5,6^ Approval for this study, including a waiver of informed consent and Health Insurance Portability and Accountability Act waiver of authorization, was obtained from the Western Institutional Review Board (protocol #20152817).

### Clinicogenomic Analyses

This study used the nationwide (US-based, ~280 US cancer clinics) de-identified Flatiron Health-Foundation Medicine PC clinico-genomic database profiled from August 2014 to September 2021. Retrospective longitudinal clinical data were derived from electronic health record (EHR) data, comprising patient-level structured and unstructured data, curated via technology-enabled abstraction, and were linked to genomic data derived from Foundation Medicine Inc (FMI) CGP tests in the FH-FMI CGDB by de-identified, deterministic matching.^7^ Outcomes of 1149 evaluable metastatic PC patients treated with 1L FOLFIRINOX or GP were examined. Real-world overall survival (rwOS) and time to next treatment (TTNT), accounting for left-truncation from start of 1L, were estimated using Kaplan-Meier analysis. Cox proportional hazards models were adjusted (aHR) for age, surgery, ECOG, CA19-9, and tissue type with random sample imputation for missing clinical values.

### Biomarkers

HRDsig was called using a machine learning-based algorithm.^4^ Copy number features and select indel features were extracted from segmented copy number profiles and used as inputs into an extreme gradient boosting (XGB) machine learning model. Training data labels were based on biallelic *BRCA1/2* as a true positive label and HRRwt (wildtype for a basket of 14 genes) as true negatives. Genome-wide focal LOH was called as previously described.^8^ HRR mutations were defined as pathogenic alterations in *BRCA1, BRCA2, ATM, BARD1, BRIP1, CDK12, CHEK1, CHEK2, FANCL, PALB2, RAD51B, RAD51C, RAD51D* and *RAD54L*.^9^ Biallelic calls were made using a computational zygosity prediction algorithm.^10^ Biallelic calls were made for cases of (1) a deep deletion in the gene, (2) 2 or more pathogenic alterations in the same gene, and (3) a pathogenic short variant under LOH. Monoallelic alterations were single-short variant alterations that were called heterozygous. In cases where zygosity could not be assessed (eg, for rearrangements or in cases where the zygosity call could not be determined), an allelic status of “unknown” was assigned.

## Results

**Figure 1.**
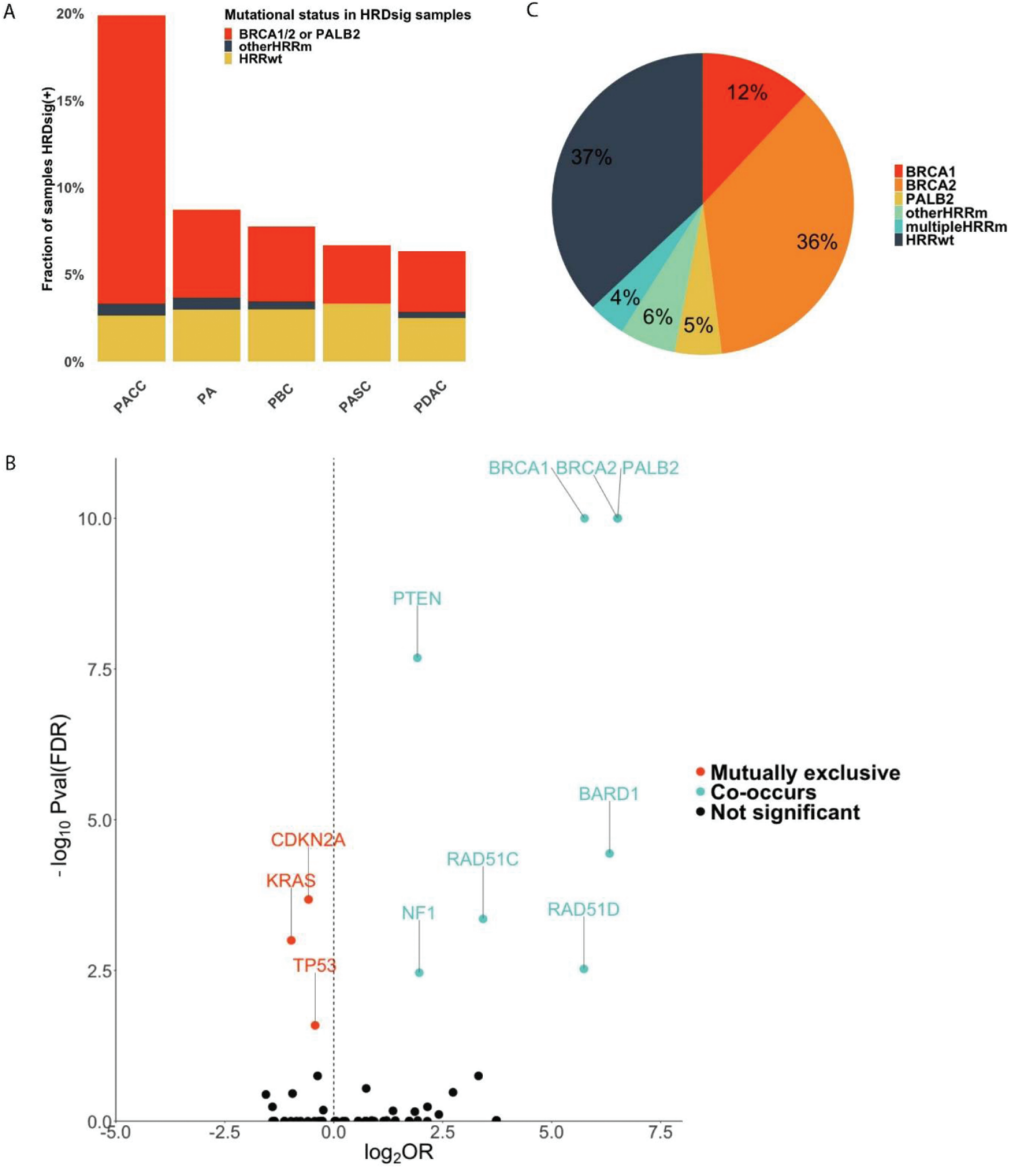
Prevalence of HRDsig in pancreatic cancer. (**A**) Prevalence of HRDsig across pancreatic cancer disease ontologies, limited to diseases with at least 50 samples. (**B**) Volcano plot examining the overlap of HRDsig with biallelic alterations in each baited gene; 2-tailed fisher’s exact *P*-values were corrected for multiple hypothesis testing using the FDR method. *P*-values were capped at 1E-10. (**C**) Pie chart examining the genomic landscape of HRDsig(+) specimens; see methods for the list of 14 HRR genes. Abbreviations: PA, pancreas carcinoma; PACC, pancreas acinar cell carcinoma; PASC, pancreas adenosquamous carcinoma; PBC, pancreaticobiliary carcinoma; PDAC, pancreas ductal adenocarcinoma.

We examined the rates of HRDsig positivity across PC tumor types and overlap with alterations in HRD-associated genes in a large real-world genomic database of PC patients (*n* = 8358). The highest rate of HRDsig positivity was observed in pancreas acinar cell carcinoma (19.9%; 30/151) and similar rates were observed across other subtypes evaluated including pancreas ductal adenocarcinoma (6.3%; 375/5938) (Fig. 1A; Supplementary Table S1). As expected, HRDsig co-occurred with biallelic alterations in *BRCA1*, *BRCA2, PALB2*, *BARD1, RAD51C,* and *RAD51D* (all *P* < .01; Fig. 1B; Supplementary Table S2) although 37% of HRDsig(+) samples were wildtype for a basket of 14 homologous recombination repair (HRR) genes (Fig 1C; Supplementary Table S3), suggesting non-genomic mechanisms of HRD. Significant overlap was observed between HRDsig and biallelic mutations in *BRCA1/BRCA2/PALB2* (Fig. 1A). There were no associations with HRDsig and monoallelic alterations in these genes (Supplementary Fig. S1). When co-occurrence analyses were rerun in the *BRCA1/2/PALB2*wt population, we observed an enrichment of biallelic alterations in other HRR genes (*RAD51C*, *RAD51D*, and *BARD1*). HRDsig was mutually exclusive with alterations in *CDKN2A*, *KRAS*, and *SMARCA4* and co-occurrent with alterations in *PTEN*, *PIK3R1*, *BRAF*, *NF2*, and *FGF10/12*, among other genes, suggesting that the propensity to have genomic scarring may differ based on molecular subtype and genomic context (Supplementary Fig. S2).

**Figure 2.**
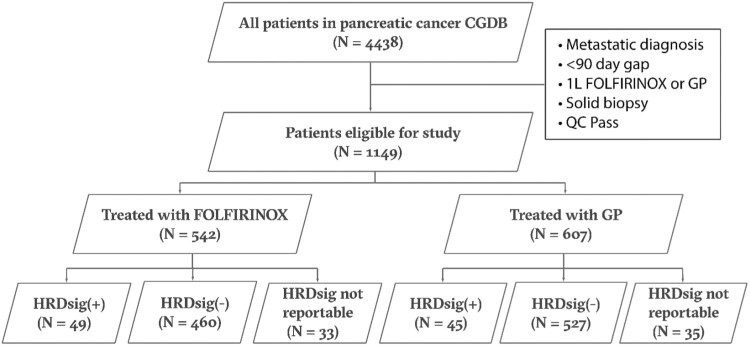
Consort diagram describing the eligible patients in the CGDB cohort.

**Figure 3.**
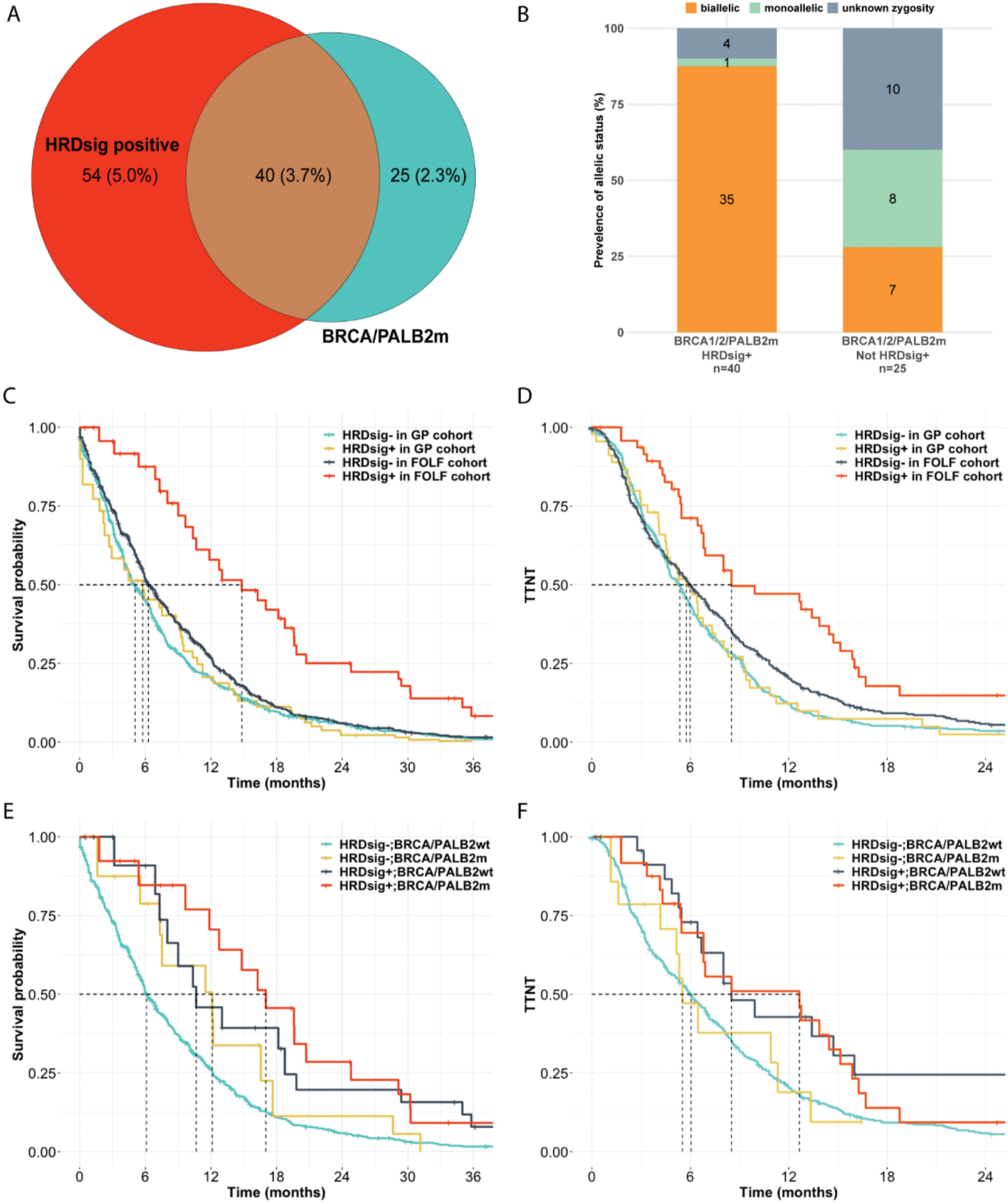
HRDsig is associated with FOLFIRINOX benefit in a clinical cohort. (**A**) Overlap of HRDsig with mutations in *BRCA1/2* or *PALB2* in the CGDB cohort. (**B**) Allelic status for *BRCA1/2/PALB2* mutant samples based on overlap with HRDsig. Kaplan-Meir plots for rwOS (**C**) and TTNT (**D**) in HRDsig(+) and HRDsig(−) patients treated with FOLFIRINOX or GP. rwOS (**E**) and TTNT (**F**) for FOLFIRINOX or GP-treated patients based on HRDsig and *BRCA1/2/PALB2* mutation status.

A total of 1149 metastatic PC patients were treated with first-line (1L) FOLFIRINOX (52%) or GP (48%) and had outcomes available for assessment as part of a de-identified clinicogenomic database (CGDB), which represents a subset of the genomic database (Fig. 2). Nine percent (94/1081) of evaluable PC patients in the CGDB were HRDsig(+), and the HRDsig+ cohort encompassed a larger population than that defined by *BRCA1/BRCA2/PALB2* mutation (9% vs. 6% of PC cases in the CGDB) (Fig. 3A). *BRCA1/2/PALB2* mutant cases that were HRDsig+ tended to be biallelic (87.5%; 35/40) while the HRDsig− cases had lower rates of biallelic status (28%, 7/25) (Fig. 3B). Patient characteristics were generally well-matched between HRDsig(+) and HRDsig(−) patients with similar age, sex distribution, race, stage, ECOG, CA19-9, and smoking history status; HRDsig(+) patients were more likely to present with liver disease (Table 1). This was in contrast to the demographic differences between the overall population of patients treated with FOLFIRINOX and GP (Supplementary Table S4). Patients treated with FOLFIRINOX were younger and had a lower ECOG status, consistent with clinical use in patients that can better tolerate these therapies.

**Table 1.** Clinical features of HRDsig(+) and HRDsig(−) patients.

	HRDsig+ (*N* = 94)	HRDsig− (*N* = 987)	*P*-value
Age at treatment start, years, median (range)	67(33-83)	66(30-95)	.611
First-line treatment			.359
FOLF	49(52.1)	460(46.6)	
GP	45(47.9)	527(53.4)	
Sex			.436
Female	46(48.9)	436(44.2)	
Male	48(51.1)	551(55.8)	
Race			.426
White	70(74.5)	648(65.7)	
Black or African American	≤5	80(8.1)	
Asian	≤5	18(1.8)	
Other race	14(14.9)	156(15.8)	
Missing	≤5	85(8.6)	
Advanced stage at diagnosis (IV)			.185
Yes	72(76.6)	712(72.1)	
No	16(17.0)	237(24.0)	
Unknown/not documented	6(6.4)	38(3.9)	
Surgery			.3788
Yes	15(17.0)	212(21.5)	
No/unknown	78(83.0)	775(78.5)	
ECOG			.31
0	34(36.2)	296(30.0)	
1	28(29.8)	366(37.1)	
≥2	8(8.5)	84(8.5)	
Missing	24(25.5)	241(24.4)	
CA19-9			.256
Normal	≤5	99(10)	
<59xULN	32(34.0)	286(29.0)	
>59xULN	27(28.7)	285(28.9)	
Missing	30(31.9)	317(32.1)	
Practice type			.028
Academic	17(18.1)	100(10.1)	
Community	77(81.9)	887(89.9)	
Biopsy site			<.001
Liver	71(75.5)	496(50.3)	
Other	8(8.5)	193(19.6)	
Pancreas	15(16.0)	298(30.2)	
Smoking status			.9531
History of smoking	50(53.2)	526(53.3)	
No history of smoking	44(46.8)	460(46.6)	
Unknown/not documented	0(0.0)	≤5	

Overall, median real-world overall survival (rwOS) for PC patients in the CGDB treated with FOLFIRINOX and GP was 6.9 and 5.1 months, respectively. Stratifying results by HRDsig status identified significantly longer rwOS and time to next treatment (TTNT) in HRDsig(+) patients treated with FOLFIRINOX relative to GP (median rwOS 14.8 vs. 5.8 months; aHR: 0.37 (0.22-0.62), *P* < .001; TTNT 8.51 vs. 5.75 mo; aHR: 0.48 (0.30-0.78), *P* < .003) (Fig 3C, 3D). In contrast, less benefit was observed for FOLFIRINOX relative to GP in the HRDsig(−) population (median rwOS 6.28 vs. 5.06 months; aHR: 0.86 (0.74-1.00), *P* = .05; TTNT 5.98 vs. 5.36 months; aHR: 0.82 (0.71-0.95), *P* = .006) (Fig 3C, 3D). rwOS at 1 and 2 years was 58% and 25% in FOLFIRINOX-treated vs. 21% and 2% in GP-treated HRDsig(+) patients. While FOLFIRINOX and GP are the most commonly used chemotherapy regimens in the first-line setting, we examined if HRDsig had predictive value for patients treated with non-FOLFIRINOX platinum therapy (Supplementary Fig. S3). We observed a significant association with improved overall survival (median rwOS 18.6 vs. 5.7 mo in HRDsig(+) vs HRDsig(−); aHR: 0.26 (0.11-0.63); *P* = .003) and a trend toward improved TTNT (aHR = 0.61, *P* = .15).

Since *BRCA1/2/PALB2* alteration status is a biomarker for FOLFIRINOX selection in NCCN guidelines, we examined the predictive value of HRDsig in patients with and without alterations in *BRCA1/BRCA2/PALB2.* HRDsig remained predictive of rwOS in patients harboring *BRCA1/2/PALB2* alterations (FOLFIRINOX median rwOS 17.0 vs. 12.1 mo in HRDsig(+) vs. HRDsig(−), aHR: 0.17 (0.05-0.63), *P* = .007; TTNT 12.7 vs. 5.52; aHR: 0.30 (0.11-0.80), *P* = .02; nHRDSig+: 24, nHRDsig−: 15) as well as in patients wildtype for *BRCA1/2/PALB2* (FOLFIRINOX rwOS 10.6 vs. 6.1 mo in HRDsig(+) vs. HRDsig(−), aHR: 0.45 (0.26-0.78), *P* = .004; TTNT 8.51 vs. 6.05 mo; aHR: 0.51 (0.31-0.83), *P* = .007; nHRDSig+: 25, nHRDsig-: 445) (Fig 3E, 3F). These results, based on a small number of patients, suggest that HRDsig and *BRCA1/2/PALB2* mutation status are both informative for FOLFIRINOX selection. As such, selection of patients for FOLFIRINOX based on either HRDsig(+) or *BRCA1/2/PALB2* mutation status captures 11% of the evaluable PC population, compared to just 6% of the population based on *BRCA1/2/PALB2* alone. HRD positivity as defined as either HRDsig(+) or *BRCA1/2/PALB2* mutations is associated with significantly longer rwOS and TTNT on FOLFIRINOX relative to GP (median rwOS 12.8 vs. 4.5 months; aHR: 0.38 (0.24-0.61), *P* < .001; TTNT 8.5 vs. 5.3 mo; aHR: 0.52 (0.34-0.81), *P* = .003) and less FOLFIRINOX benefit in the dual-negative population (median rwOS 6.1 vs. 5.1 months; aHR: 0.88 (0.76-1.02), *P* = .10; TTNT 6.1 vs. 5.4 mo; aHR: 0.83 (0.72-0.95), *P* = .009).

## Discussion

In summary, our study identified HRDsig positivity as a frequent event in PC, impacting nearly 1 in 10 patients. Based on an evaluation of rwOS and TTNT, this scar-based measure of HRD was predictive of significant benefit on platinum-containing FOLFIRINOX relative to GP while less benefit from FOLFRINOX was observed in an HRDsig(−) population. HRDsig complements *BRCA1/2/PALB2* mutation status, identifying a larger population of patients and maintaining predictive value in both wildtype and mutant cohorts.

The choice of 1L standard of care therapy in PC is challenging. The decision to prescribe GP or FOLFIRINOX comes with trade-offs, and as evidenced in our real-world CGDB these regimens are being prescribed at comparable frequencies in the 1L setting. While some patients will obtain significant benefits from FOLFIRINOX, high-grade toxicities including neutropenia, fatigue, vomiting, and diarrhea occur frequently with potential impact on patient quality of life, thus biomarkers to identify patients who do not derive significant added OS/TTNT benefit from FOLFIRINOX are valuable. Our study identified significant associations of FOLFIRINOX OS/TTNT in patients with *BRCA1, BRCA2,* or *PALB2* alterations. HRR alterations, especially those in *BRCA1, BRCA2,* and *PALB2* have been associated with sensitivity to platinum agents in ovarian and breast cancer.^11,12^ Our findings are consistent with a small study of 26 patients harboring germline mutations in *BRCA1, BRCA2,* or *PALB2* which identified a higher response rate and rwPFS on FOLFIRINOX relative to a wildtype control group.^3^

This study suggests the added value of HRDsig beyond these genomic biomarkers, providing potential value in 1L PC treatment choice regardless of HRR mutational status. Patients negative for HRDsig or dual-negative for HRDsig and BRCA1/2/PALB2 alterations obtained less benefit from FOLFIRINOX vs. GP (median rwOS 6.1 vs. 5.1 mo; *P* = .10), suggesting that GP may be a more favorable treatment option in these patients to minimize toxicities. Conversely, we showed significantly more benefit for FOLFIRINOX vs. GP in patients positive for HRDsig or dual-positive for HRDsig(+) and/or *BRCA1/2/PALB2*. When FOLFIRINOX can be tolerated, this treatment may be preferable as many HRDsig(+) patients exhibited long rwOS intervals, including 25% of patients alive at 2 years.

**Figure 4.**
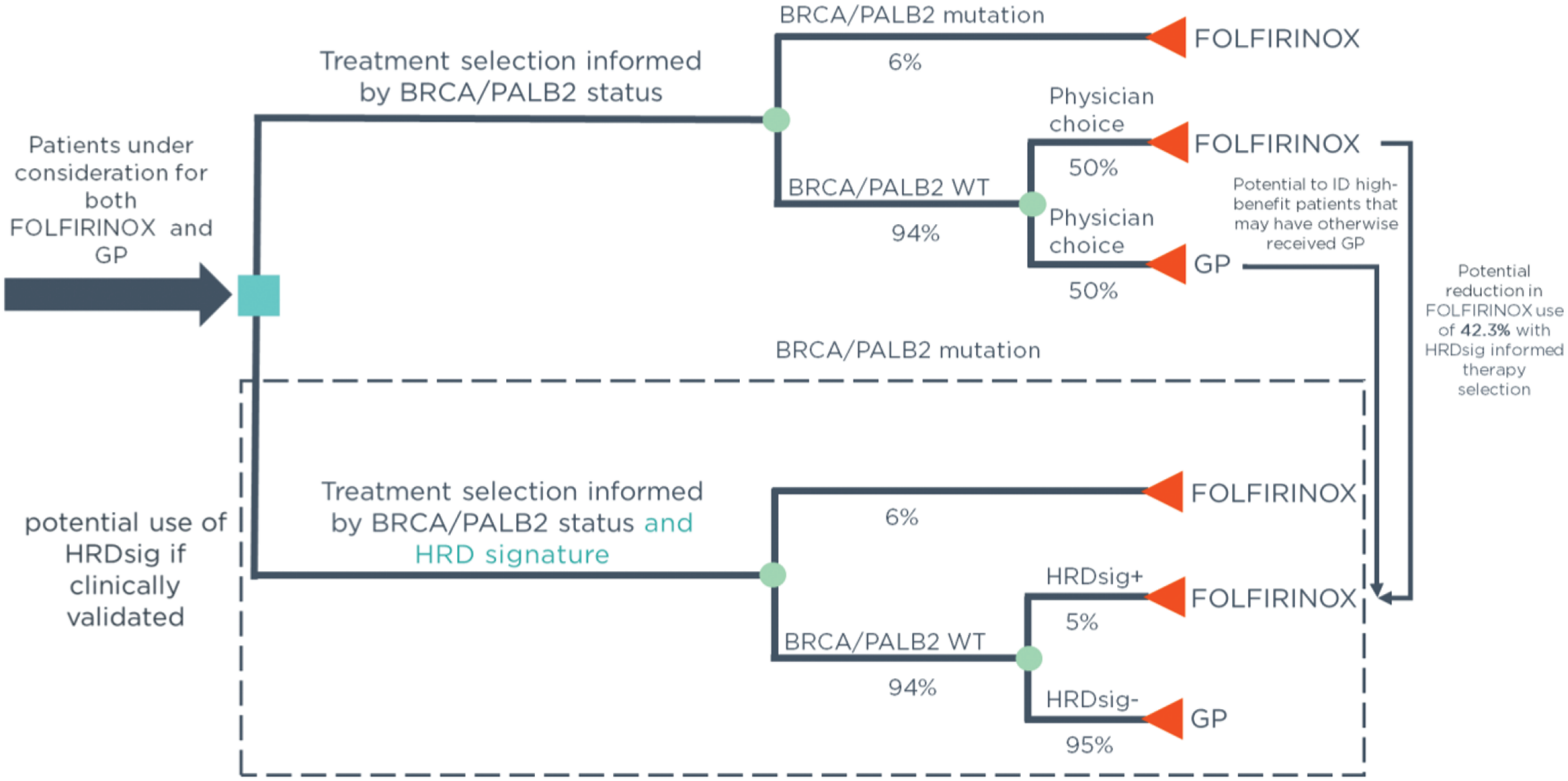
Potential implications of HRDsig on the pancreatic cancer decision landscape. The current treatment paradigm, selecting patients for FOLFIRINOX or GP based on *BRCA1/2* or *PALB2* mutation status is shown on the top branch. Patients negative for the biomarker (94% of patients) will receive physicians’ choice FOLFIRINOX vs. GP. If a scar-based biomarker of HRD is clinically validated, its use could identify additional patients who might benefit from FOLFIRINOX (bottom branch).

In the front-line setting, the current treatment paradigm for pancreatic cancer provides decision support only in the cases of BRCA1/2 or PALB2 alterations (Fig. 4 top branch). For the ~94% of patients negative for this biomarker, physicians must weigh the tradeoffs of toxicity and perceived patient-level benefit. If scar-based biomarkers like HRDsig are clinically validated, they can aid in personalized therapy selection and could improve patient care in 2 ways (Fig. 4 bottom branch). First, it could enable FOLFIRINOX selection for patients who may benefit from the therapy. Under the current paradigm, ~2.4% of patients may receive GP even when they are HRDsig(+) and may benefit from FOLFIRINOX (Fig 4; Supplementary Fig S4). Second, our retrospective study suggests that HRDsig-negative and/or *BRCA1/2/PALB2* negative patients have less benefit on FOLFIRINOX. Prospective studies should examine if a subset of patients have acceptable outcomes on GP alone, potentially allowing for significant reductions in adverse events, toxicity, and hospitalizations. In the ACCORD11/PRODIGE4 trial, high rates of grade 3/4 adverse events were observed in the FOLFIRINOX arm, including 46% with neutropenia, 24% with fatigue, 14.5% with vomiting, and 13% with diarrhea, impacting both patient quality of life and hospitalization related health system costs.^13^ A clinically validated scar-based HRD biomarker could potentially reduce FOLFIRINOX use (Fig. 4; Supplementary Fig. S4).

An important practical consideration, in the use of NGS scar-based biomarkers is the availability of results when making a first-line treatment decision. In our cohort, for the physicians who received CGP within 30 days of advanced diagnosis, only 23% waited to start treatment until after CGP results were obtained. CGP results have received a median of 9 days after the start of first-line therapy. Delayed ordering of CGP following diagnosis, or unwillingness or inability to wait for CGP results before start of therapy have the potential to significantly attenuate utility in the first line. As the clinical utility of HRDsig in pancreatic cancer becomes more established, reflex ordering for HRDsig would dramatically decrease the turnaround for CGP testing, and physicians would be better equipped to make an informed treatment decision and fulfill the promise of precision oncology for pancreatic cancer patients. In lung cancer, timely CGP to inform first-line therapy is associated with increased use of precision therapy including genomically matched targeted therapies, although the authors acknowledge that in some cases immediate start of therapy without CGP results is necessary (ESMO 2021 1301P).^14^ It is an open question whether scar-based biomarkers may also have utility in the second-line setting in pancreatic cancer as this study was limited to the first line.

Wainberg et al recently reported on the results from NAPOLI-3, a phase 3 trial examining liposomal irinotecan + 5-fluorouracil/leucovorin + oxaliplatin (NALIRIFOX) vs. GP in first-line mPDAC. Without using any biomarker stratification, the authors observed significantly better OS and PFS in the NALIRIFOX arm (OS HR = 0.84, *P* = .04; PFS HR = 0.70, *P* = .0001).^15^ These results suggest potential broad value in an unselected population. We observed significant associations of HRDsig status with rwOS and a trend toward association with TTNT for non-FOLFIRINOX platinum regimens. However, it is unknown how HRDsig might stratify responses in patients treated with NALIRIFOX v GP.

PARP inhibitors (PARPi) were recently approved in the maintenance setting for patients with deleterious germline *BRCA1/2* alterations who have not progressed on 1L platinum chemotherapy regimens based on the results of the POLO trial.^16^ Since we identify a set of HRDsig(+) patients, which includes a subset of *BRCA1/BRCA2/PALB2* wildtype patients, with durable responses to platinum-containing chemotherapy, it is tempting to speculate on possible response to other HRR-targeted therapies, including PARPi. Future trials should examine the potential value of PARPi in the maintenance setting for HRDsig(+) patients who have responded to FOLFIRINOX.

Our study was limited in that it relied on a real-world dataset. Randomized control trials provide the highest level of evidence by minimizing cohort differences between arms. However, in this study clinical characteristics were similar between groups. Results were significant in univariate and multivariate analyses, after adjusting for relevant clinical characteristics including age, ECOG status, and CA19-9. While the cohort was large at >1100 patients, some subset analyses were underpowered. Additional validation of our findings in an independent cohort would strengthen these results. An additional limitation of this study are the clinical endpoints. Associations of HRDsig were shown for TTNT and rwOS, however, information is not available on the clinical benefit rate (eg, CR, PR, and SD).

## Conclusion

In summary, our study found that a novel scar-based HRD signature identifies a subset of patients with exceptional benefit on FOLFIRINOX with more than doubled median rwOS and with a substantial population alive at 2 years (25%), and with predictive value in both the BRCA/PALB2 mutant and wildtype settings. This work underscores the need to develop and validate scar-based HRD measures that will complement genomic approaches for the selection of 1L chemotherapy and possibly for selection of PARPi in pancreatic cancer. Additional studies are urgently needed to address this poor-prognosis disease.

## Supplementary Material

oyad178_suppl_Supplementary_Figures

oyad178_suppl_Supplementary_Tables

## Data Availability

The sequencing data generated in this study is derived from clinical samples. The data supporting the findings of this study is provided within the paper and its supplementary files. All supplementary information accompanying the different analyses and figures presented in this study are provided in the Supplementary material files. Due to HIPAA requirements, we are not consented to share individualized patient genomic data, which contains potentially identifying or sensitive patient information. Foundation Medicine is committed to collaborative data analysis, and we have well-established, and widely used mechanisms by which investigators can query our core genomic database of >700 000 de-identified sequenced cancers to obtain aggregated datasets. Requests for collaborative data shares can be made by contacting the corresponding author(s) and filling out a study review committee form. Once approved, investigators are required to sign a data transfer agreement. Written proposals are considered at monthly meetings and data transfer agreements expire 18 months from execution of the agreement.
