# Supplementary material for: A Novel HRD Signature Is Predictive of FOLFIRINOX Benefit in Metastatic Pancreatic Cancer
Source: Oncologist. 2023 Jun 24;28(8):691–8. doi: 10.1093/oncolo/oyad178 (PMC10400136; doi:10.1093/oncolo/oyad178)
Supplement: oyad178_suppl_Supplementary_Figures [file oyad178_suppl_supplementary_figures.docx]

**Supplemental Figures**

**Figure S1. Association of HRDsig with monoallelic alterations.** Volcano plot examining the overlap of HRDsig with monoallelic alterations in each baited gene; two-tailed fisher’s exact p-values were corrected for multiple hypothesis testing using the FDR method.

**
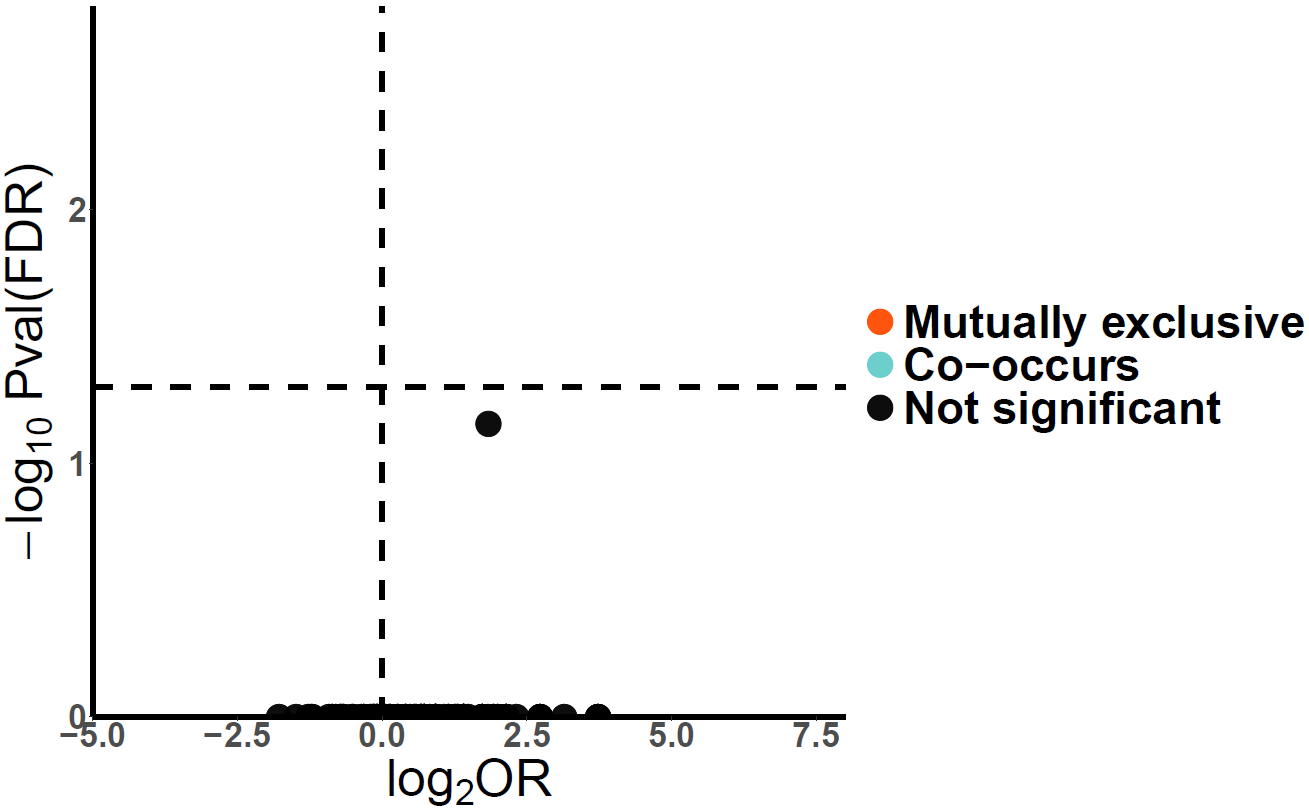
**

**Figure S2. Co-occurrence analyses in the BRCA1/2/PALB2wt population.** (A) Overall co-occurrence (B) Co-occurrence with biallelic alterations. P-values were capped at 1E-5 and log2 OR were capped at +/- 5.
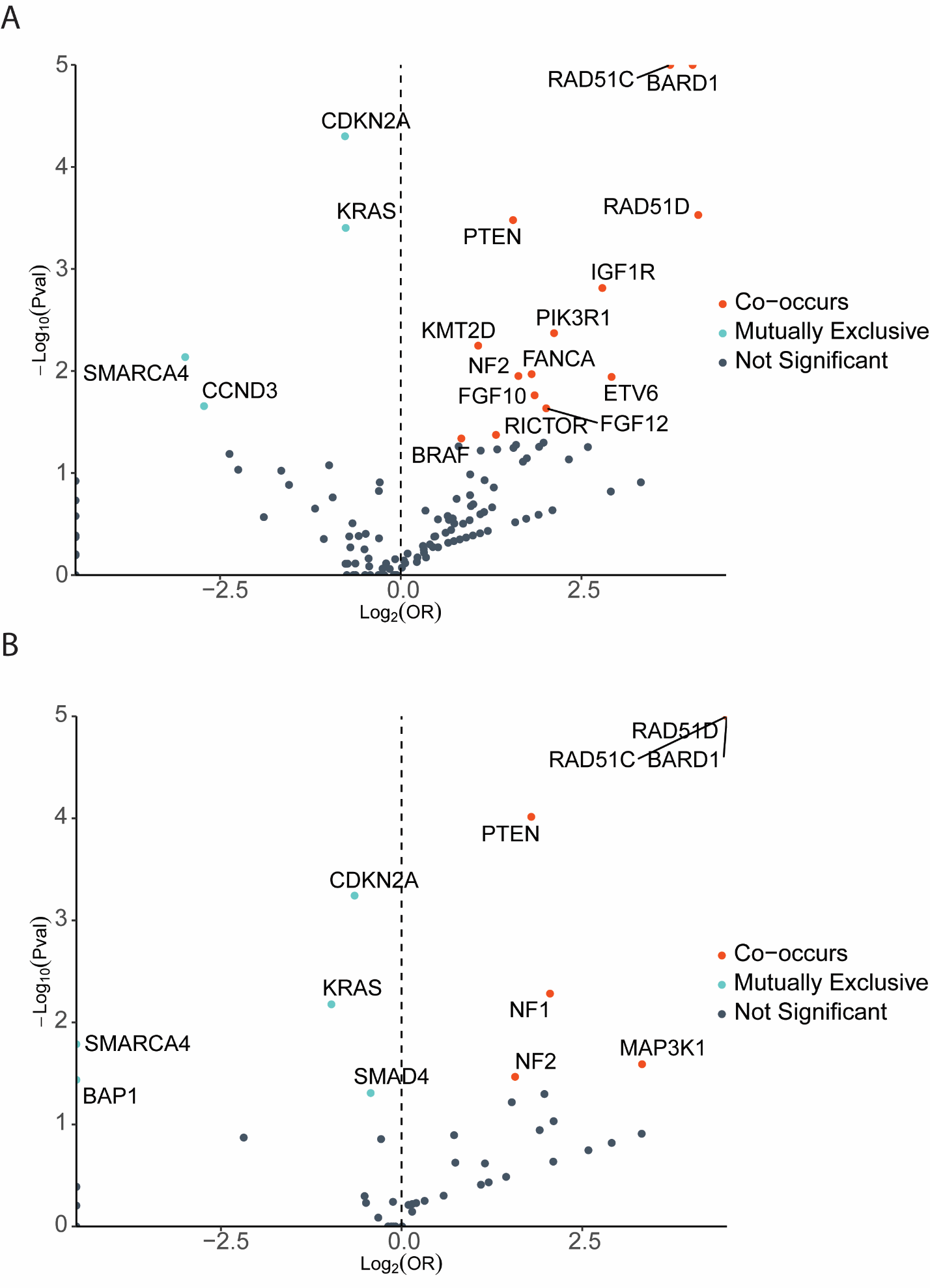


**Figure S3. Association of HRDsig with rwOS (A) and TTNT (B) for patients treated with 1^st^ line non-FOLFIRINOX platinum regimens.**

**
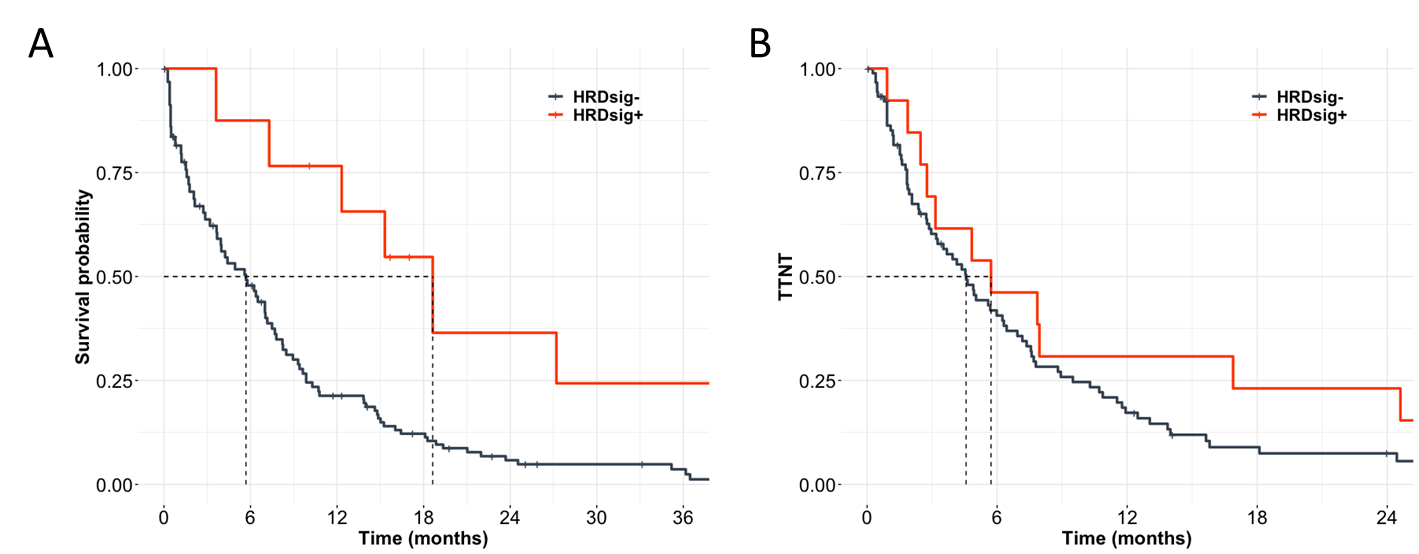
**

**Figure S4. Potential implications of HRDsig on the pancreatic cancer decision landscape.**

**
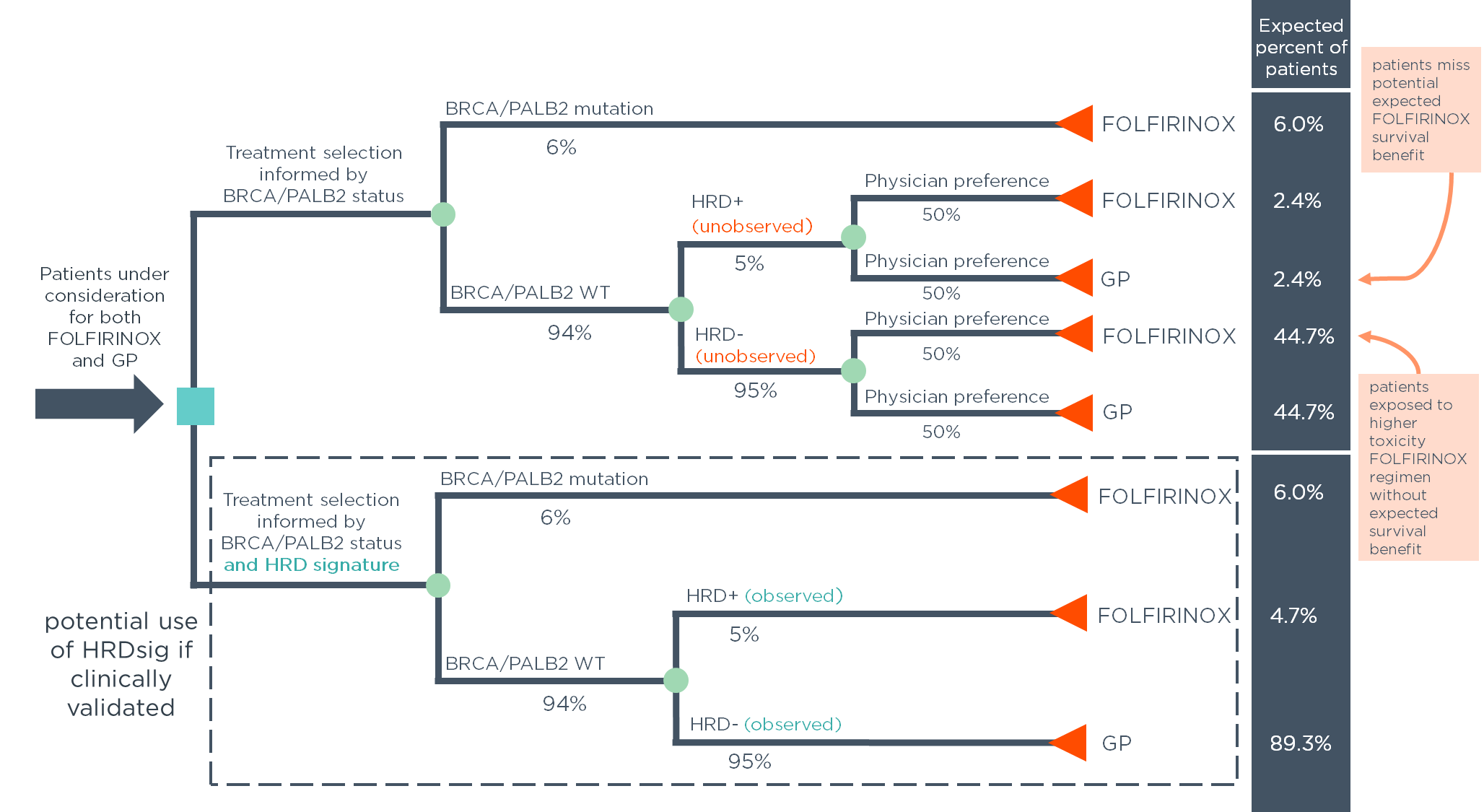
**
